# Protein Kinase C‐Delta (PKCδ) inhibition stabilizes endothelium and suppresses triple‐negative breast cancer (TNBC) intravasation in a microfluidic hypoxic tumor model

**DOI:** 10.1002/btm2.70090

**Published:** 2025-11-18

**Authors:** Indira Sigdel, Awurama Ofori‐Kwafo, Earshed Al Mamun, Amit K. Tiwari, Yuan Tang

**Affiliations:** ^1^ Department of Bioengineering, College of Engineering The University of Toledo Toledo Ohio USA; ^2^ Department of Pharmaceutical Sciences, College of Pharmacy University of Arkansas for Medical Sciences Little Rock Arkansas USA

**Keywords:** endothelial cells, hypoxia, inflammation, metastasis, microfluidics, triple‐negative breast cancer, tumor microenvironment

## Abstract

Metastasis is the principal cause of mortality in breast cancer, but therapies specifically targeting metastatic mechanisms are scarce. In triple‐negative breast cancer (TNBC), hypoxia within the tumor microenvironment (TME) promotes endothelial dysfunction, increasing vascular permeability and facilitating cancer cell intravasation. This study presents a microfluidic‐based idealized microvascular on‐chip (iMVoC) model utilizing human umbilical vein endothelial cells and TNBC cells (SUM159PTX) to model a hypoxic TME. This model mimicked dynamic flow perfusion, promoting endothelial alignment along the flow direction, while supporting 3D tumor structures exhibiting varying oxygen levels in the tissue compartment. The iMVoC model enabled cell–cell interactions and the exchange of media and nutrients between compartments. Hypoxia was confirmed by increased nuclear translocation of hypoxia inducible factors (HIF)‐1α and HIF‐2α in TNBC cells, indicating hypoxia‐based signaling. Hypoxia‐induced endothelial cell (EC) inflammation was validated through elevated permeability, upregulation of adhesion molecules, and increased reactive oxygen species (ROS) production, suggesting activation of the HIF‐ROS pathway. Enhanced tumor cell intravasation was observed across inflamed endothelium, and cytokine profiling further confirmed EC activation through inflammatory signaling. Application of the protein kinase C delta (PKCδ) inhibitor (PKCδ‐TAT) significantly mitigated these effects, shifting HIF localization from the nucleus to the cytoplasm, reducing ROS production, downregulating inflammatory cytokines, and lowering TNBC intravasation. These findings demonstrate PKCδ as a key mediator linking hypoxia to EC dysfunction and tumor dissemination. Protecting EC barrier integrity emerges as a promising strategy to mitigate hypoxia‐driven TNBC metastasis, with the iMVoC platform offering a valuable tool for testing anti‐cancer therapeutics or drug combinations involving PKCδ‐TAT.

**Translational Impact Statements**

We developed a microvascular‐on‐chip model that simulates hypoxic tumor–endothelial interactions to study mechanisms of metastasis. Our findings reveal that targeting protein kinase C delta protects vascular integrity and reduces triple‐negative breast cancer cell intravasation under hypoxic conditions. This work highlights the translational potential of biomimetic microfluidic platforms for identifying therapeutic strategies aimed at limiting metastasis in aggressive cancers.

## 1 INTRODUCTION

Endothelial cells (ECs) maintain vascular integrity and contribute to physiological processes, including homeostasis, development, organ formation, nutrient and waste transfer and responses to pathological events.^1^, ^2^ Under normal conditions, ECs are continuously subjected to mechanical stimulation from blood flow (shear stress), which influences their shape and function,^3^ however under pathological conditions like cancer, their dysfunction directly contributes to tumor progression and metastasis.^4^, ^5^ Metastasis, a multi‐step process where cancer cells escape from the primary site, survive in the bloodstream, and seed on the secondary site and proliferate,^6^ is greatly influenced by the tumor microenvironment (TME), a complex environment housing cancer cells along with other supporting cells and soluble signaling molecules.^7^ ECs, which form the barrier between blood circulation and surrounding tissue, influence the metastatic steps (intravasation and extravasation) through interactions between cancer cells and ECs, acting as entry and exit points for metastasizing cancer cells.^8^ The integrity of the endothelial barrier is often compromised in tumors due to molecular and phenotypic changes in ECs.^9^, ^10^, ^11^ Disseminating cancer cells secrete inflammatory mediators that directly induce EC activation, leading to enhanced vessel permeability^12^; meanwhile, inflammatory mediators upregulate the expression of adhesion molecules on the EC surface and ligands on the circulating cancer cells that recognize them,^13^, ^14^ resulting in increased intravasation and extravasation of tumor cells. This compromise allows cancer cells to breach the vasculature, enter circulation, and ultimately establish secondary tumors at distant sites.^9^, ^10^

Triple‐negative breast cancer (TNBC), among other subtypes, stands out as the clinical case where the metastatic progression poses a challenge^15^ and metastasis accounts for approximately 90% of breast cancer‐related mortality.^16^ TNBC contributes to its early dissemination to other distant organs, making effective management of its metastatic potential an unmet clinical need and this metastatic behavior is largely influenced by its TME which is similar to the normal tissue microenvironment, but differs in terms of oxygen levels.^17^ As solid tumors grow, they quickly outpace the formation of new blood vessels, leading to regions of low oxygen, known as hypoxia.^17^ In response, cancer cells express hypoxia inducible factors (HIFs), proteins that promote cell adaptation in low‐oxygen conditions through molecular and phenotypic changes, including the induction of HIF transcription factors.^18^ HIFs are composed of oxygen‐sensitive subunits HIF‐α (HIF‐1α, HIF‐2α, and HIF‐3α) and constituently express HIF‐1β subunits.^19^ Under normal oxygen conditions, HIF‐α are degraded; however, under hypoxic stress, HIFs are stabilized, as they translocate from cytoplasm to nucleus where it binds with its counterpart HIF‐1β, activating the transcription of various downstream genes. Hypoxia, defining characteristic of most solid tumors, is closely linked to cancer progression, immune evasion, therapy resistance, and metastasis^20^ by stabilizing HIFs, which reprogram cells to adapt through angiogenesis, metabolic changes, and increased invasiveness. Hypoxic signaling promotes the early stages of metastasis associated with tumor cell invasion, migration, and intravasation.^21^ Hypoxic stress profoundly alters EC function, triggering endothelial activation, increasing vascular permeability, and promoting a pro‐inflammatory, pro‐metastatic phenotype that facilitates tumor cell intravasation.^22^ Hypoxia in the TME also triggers inflammatory responses through the activation of HIFs, which promotes the release of pro‐inflammatory mediators and growth factors that fosters immune cell infiltration, tumor immune evasion, and EC activation.^22^, ^23^ This inflammation contributes to the tumor growth and influences the behavior of surrounding stromal cells, particularly ECs, making them more permissive to cancer cell intravasation. Tumor‐associated inflammation has been shown to work in synergy with hypoxia to increase endothelial permeability and establish a supportive vascular niche for metastasis.^23^, ^24^ Together, hypoxia and inflammation work together, setting the stage for metastasis, and understanding their interactions within TME is important in developing effective treatment strategies.

A significant consequence of chronic inflammation in tumors is the production of reactive oxygen species (ROS), which act as both signaling molecules and sources of oxidative stress.^24^, ^25^ Increased levels of ROS lead to ECs dysfunction by disrupting endothelial junctions, elevating adhesion molecule expressions, and compromising the vascular barrier, all of which facilitate cancer cell migration into circulation.^24^, ^26^ This hypoxia‐induced inflammation creates a feedback loop via the HIF‐NF‐κB axis that drives both EC activation and TNBC intravasation. Among the downstream effectors of EC activation caused by hypoxia and inflammation is protein kinase C delta (PKCδ), an important signaling molecule. Oxidative stress and pro‐inflammatory cytokines within the hypoxic TME cause PKCδ to become activated, increasing endothelial permeability by altering cytoskeletal dynamics, breaking down endothelial junctions and upregulating the expression of adhesion molecules on the surface of ECs. PKCδ works by weakening or damaging the barrier integrity, eventually permitting cancer cells to intravasate through these mechanisms.

Previously,^27^ using our idealized microvascular on‐chip (iMVoC) microfluidic model, which is a transparent polydimethylsiloxane (PDMS)‐based device featuring a central tissue compartment flanked by perfusable vascular channels separated by a porous microfabricated interface, we created a tumor–endothelial co‐culture system to assess the anti‐cancer efficacy of thienopyrimidine analogs on TNBC cells. Building on that foundation, the present study introduces a hypoxic iMVoC model with structural and functional improvements that extend its application from compound testing to mechanistic interrogation of hypoxia‐driven endothelial dysfunction and TNBC intravasation. This refined model incorporates primary human ECs and metastatic TNBC cells in a dynamic flow perfusion system, successfully simulating in vivo vascular conditions and endothelial barrier function. Our findings highlight the significance of preserving endothelial integrity in restricting TNBC metastasis. It remains unclear to what extent tumor‐activated ECs contribute passively through structural abnormalities versus actively regulating cancer cell intravasation via hypoxia‐ and inflammation‐induced signaling pathways. Here, we investigate whether PKCδ inhibition can stabilize the endothelial barrier and suppress hypoxia‐induced TNBC intravasation. Using a physiologically relevant hypoxic iMVoC model that replicates dynamic perfusion, tumor–EC interactions, and oxygen gradients, we examine the role of PKCδ in mediating endothelial dysfunction under hypoxic conditions. Our findings reveal that hypoxia‐induced EC activation leads to increased vascular permeability, upregulation of adhesion molecules (intercellular adhesion molecule 1 [ICAM‐1], E‐selectin), and enhanced tumor cell intravasation. Elevated ROS further contribute to endothelial dysfunction, amplifying metastatic potential. Inhibition of EC activation using PKCδ‐TAT, a peptide inhibitor of EC inflammation,^28^, ^29^, ^30^, ^31^ reduces hypoxia‐induced endothelial activation, preserving vascular integrity and reducing TNBC intravasation. These findings underscore the active involvement of the endothelium in TNBC metastasis and propose PKCδ inhibition as a promising therapeutic approach to counteract hypoxia‐induced endothelial dysfunction.

## 2 MATERIALS AND METHODS

### 2.1 Materials, reagents, and equipment

Human umbilical vein endothelial cells (HUVECs, CC‐2519) and Endothelial Cell Growth Medium (EGM; NC0916308) were purchased from Lonza (Basel, Switzerland). Dulbecco's Modified Eagle Medium (DMEM, 10‐013‐CV), fetal bovine serum (FBS, 35‐016‐CV), and penicillin–streptomycin (Pen‐Strep, 15140‐148), Human Plasma Fibronectin (341635), Matrigel (47743‐722), phosphate buffered saline (PBS; SH30356.01), 75 cm^2^ culture flasks (130190), 4 kDa fluorescein isothiocyanate (FITC) dextran (T1037‐100MG), 0.25% Trypsin–EDTA (25200‐056), anti‐h/m/rHIF‐1alpha/HIF1A, purified mouse monoclonal IgG (HIF‐1α, MAB1536), HIF‐2 alpha monoclonal antibody (ep190b) (HIF‐2α, MA1‐16519), goat anti‐mouse IgG (H + L) highly cross‐adsorbed secondary antibody Alexa Fluor Plus 488 (A32723), fixative solution (FB002), Triton X‐100 (648463‐50ML), normal goat serum (31872), NucBlue Live Cell Stain Ready Probes (R37605), SlowFade Gold antifade reagent (S36936), Dulbecco's phosphate buffered saline/modified (DPBS 1×; +calcium, +magnesium; SH30264.01), Hanks' balanced salt solution (HBSS), CellTracker CM‐DiI (C7001), ICAM‐1 (16‐0549‐82), E‐selectin (14‐0627‐82), immunoglobulin G1 (IgG1, 14‐4714‐82), protein A/G (21186), dimethyl sulfoxide (DMSO, CAS 67‐68‐5), and isopropanol, 70% v/v (A459‐4) were purchased from Fisher Scientific (Hampton, New Hampshire). Image iT Hypoxia red dye (H10498), 10 μm green, fluorescent polystyrene beads (G1000), Image iT LIVE Green Reactive Oxygen Species Detection Kit (I36007) and Chemokine/Cytokine proteome profiler Kits (ARY022B) were all obtained from Fisher Scientific. PKCδ‐TAT (Mimotopes, Australia) was a generous gift from Dr. Laurie Kilpatrick. The microfluidic devices used in this study were purchased from SynVivo Inc. (SynTumor 102012). Experiments were conducted using an Olympus IX71 fluorescence microscope equipped with an automated stage for precise imaging. Images were captured with an ORCA Flash 4 camera (Hamamatsu Corp., USA). Additionally, a PHD Ultra Syringe Pump (Harvard Apparatus, USA) was employed to inject growth media, permeability dye, or microbeads suspensions into the channels of the microfluidic device.

### 2.2 Cell culture

The SUM159PTX cell line was a generous gift from Dr. Amit K. Tiwari.^27^, ^32^ HUVECs were cultured using the EGM and EGM supplements kit, while DMEM supplemented with 10% FBS and 1% Pen‐Strep was used to culture cancer cells. All cells were grown in tissue culture flasks inside a 37°C incubator, maintaining 5% CO_2_ and 95% humidity. Cells were rinsed with PBS and trypsinized to dislodge from flasks and sub‐cultured when they reached 80%–90% confluency. HUVECs were used from passages one to five.

### 2.3 Establishing the hypoxic iMVoC model

A previously established protocol was utilized to establish the hypoxic iMVoC model.^27^ Briefly, the microfluidic device was degassed to remove air bubbles. HUVECs were seeded in the vascular channels, pre‐coated with fibronectin, at a density of 25 million cells/mL, and incubated for 4 h at 37°C in static conditions, followed by continuous media perfusion at a shear stress of 0.175 dyne/cm^2^. The next day, TNBC cells were resuspended in 15% Matrigel at a density of 15 million cells/mL and seeded into the central compartment, and the device was left to sit for another 48 h to form a 3D TNBC‐EC co‐culture model. The flow scheme utilized for this study was: Step 1: 0.1 μL/min for 12 h, Step 2: delay of 12 h, and Step 3: Repeat from Step 1 until the end of experiments. After 72 h of initial HUVEC seeding and 48 h of TNBC seeding, the device was used for downstream assays as described in the upcoming sections. Throughout the experiments, the vascular channels were perfused with either fresh medium or medium supplemented with PKCδ‐TAT, maintaining ECs under normoxic flow conditions.

### 2.4 Probing oxygen concentration in the hypoxic iMVoC model

To visualize and estimate oxygen levels, we used Image‐iT™ Red Hypoxia Reagent (Thermo Fisher), a fluorogenic dye that becomes fluorescent in live cells under low‐oxygen conditions (<5% O_2_). A calibration curve is generated using sodium sulfite (Na_2_SO_3_) to create oxygen‐scavenged media at defined oxygen levels, validated with a dissolved oxygen (DO) meter. These media solutions are incubated with 5 μM Image‐iT™ Red Hypoxia Reagent and live TNBC cells directly within the tissue compartment of iMVoC. After 1‐h incubation at 37°C, fluorescence images are acquired (Ex/Em: 577/602 nm) under standardized exposure settings. The fluorescence intensity is then correlated with known oxygen concentrations to establish a device‐specific standard curve, enabling spatial estimation of hypoxia levels within the model.

For hypoxia visualization in experimental conditions, 5 μM Image‐iT™ Red was introduced into the tissue compartment after TNBC seeding and incubated for 1 h. Unbound dye was rinsed off, and the device was reconnected to a syringe pump to maintain continuous perfusion through the vascular channels. Bright‐field and fluorescence images were captured every 24 h using a 10× objective. Fluorescence intensity was quantified using cellSens and MATLAB to generate pseudo‐color spatial distributions of intracellular hypoxia. While the dye's activation is cell‐dependent, this calibration enabled semi‐quantitative oxygen mapping within the iMVoC based on biological response.

### 2.5 Detecting HIF‐1α and HIF‐2α

To detect the isoforms of HIFs in the hypoxic iMVoC model, immunohistochemistry was performed on cells in all vascular channels and tissue compartments containing HUVECs and TNBC cells after 72 h of exposure to hypoxic conditions. Imaging was conducted under both untreated and PKCδ‐TAT treated conditions. Following treatment, the cells were fixed with 4% formaldehyde for 10 min at 4°C, followed by rinsing twice and permeabilized using 0.1% v/v Triton X‐100 for 10 min at 4°C. Then the cells were rinsed again and blocked with 5% v/v normal goat serum (diluted in PBS) at 37°C for 1 h. After blocking, the channels were incubated overnight at 4°C with primary antibodies (HIF‐1α, HIF‐2α). The next day, the cells were washed with PBS, followed by incubation with secondary antibodies for 1 h at 37°C. The cells were counterstained with Hoechst dye for nuclear staining and imaged using the same microscope system mentioned earlier. The presence and subcellular distribution of HIF‐α were determined via immunostaining followed by fluorescence imaging. Fluorescence images were analyzed to quantify the HIF expression levels and assess their translocation into the nucleus and cytoplasm. HIF‐α translocation was quantified by intensity analysis in ImageJ software, and the values were expressed as the ratio of nucleus intensity to cytoplasm intensity.^33^

### 2.6 PKCδ‐TAT peptide inhibitor treatment

After 72 h of HUVECs seeding, PKCδ‐TAT (5 μM^29^, ^30^, ^33^, ^34^, ^35^ in EGM) was introduced into the vascular channels using a syringe pump to ensure precise and controlled delivery, mimicking physiological exposure. Vascular channels were continuously perfused with PKCδ‐TAT for 72 h.,^29^, ^30^, ^33^, ^34^, ^35^ with images taken daily to monitor cellular responses. Following treatment, functional assays were conducted, including permeability assays, adhesion molecule expression analysis, and ROS quantification, to evaluate the effect of PKCδ inhibition on endothelial barrier integrity, inflammation, and TNBC intravasation potential.

### 2.7 Permeability measurements

To evaluate endothelial barrier integrity the permeability of endothelial barriers was assessed using a 4 kDa FITC Dextran dye. The dye was infused into the vascular channel at a rate of 1 μL/min (1.75 dyne/cm^2^) for 1 h while time‐lapse imaging was acquired every minute using the cellSens Dimension software and an Olympus microscope system. Fluorescence intensity was recorded for both the vascular and tissue compartments using drawn regions of interest (ROIs), and the difference in intensity between these compartments was used to calculate permeability following an established protocol.^27^ The permeability coefficient (*P*) was determined using the equation:

$$P=\left(1-H_{\mathrm{CT}}\right)\left(\frac{1}{I_{0}}\right)\left(\frac{\mathrm{dI}}{\mathrm{dt}}\right)\left(\frac{V}{S}\right),$$

where *H*_CT_ represents hematocrit in the vascular channel (*H*_CT_ = 0 since the EGM medium does not contain blood cells), *I* corresponds to the average intensity in the tissue compartment at a given time point, *I*_0_ denotes the maximum fluorescence intensity in the vascular channel, and *V*/*S* represents the volume‐to‐surface area ratio of the vascular channel. The obtained fluorescence intensity values were analyzed to quantify endothelial permeability.

### 2.8 Measuring endothelial adhesion molecule expression

The activation of ECs was evaluated using a polystyrene microbead adhesion molecule assay to assess the functional relevance of adhesion molecule upregulation. To conduct this experiment, on Day 1, 10‐μm fluorescent microbeads were aliquoted from the stock solution, washed twice with a sodium bicarbonate buffer, and coated with protein A/G (300 μg/mL) via passive adsorption, followed by overnight incubation at room temperature. The next day, the microbeads were washed with blocking solution (1% bovine serum albumin [BSA] in HBSS) and incubated for 30 min at room temperature, during which microbeads were counted. After incubation, the suspension was centrifuged, and the supernatant was removed before antibody addition. ICAM‐1, or E‐selectin antibodies (0.1 mg/mL) were added to the 5 × 10^6^ beads/mL and incubated for 1 h at 37°C. The prepared beads could be stored for up to 7 days at 4°C. Before using the devices, microbeads were washed with HBSS to remove unbound antibodies. IgG1‐coated microbeads were prepared using the same protocol to ensure specificity in adhesion molecule detection and served as a negative control. Following antibody coating, the microbeads were resuspended in EGM media and introduced into microfluidic devices containing EC monolayers. The flow rate was maintained at ~1.75 dyne/cm^2^ for 60 min to allow adhesion interactions to occur. After incubation and washing off unbound microbeads, adherent ones were imaged via fluorescence microscopy and quantified using cellSens Dimension software.

### 2.9 Detecting reactive oxygen species generation

To assess the role of ROS in the hypoxic iMVoC model, the ROS Detection Kit was utilized which incorporated a cell‐permeable fluorescent dye that remains non‐fluorescent under reduced conditions but fluoresces upon oxidation by ROS, enabling real‐time visualization and quantification of ROS production. For ROS detection, TNBC cells were labeled with Carboxy‐H2DCFDA at a working concentration of 25 μM. The TNBC cells were washed with HBSS, followed by labeling using prepared solutions and incubating the devices for 30 min at 37°C, protected from light. After incubation, the cells were washed with HBSS before imaging. The Olympus IX71 fluorescent microscope with FITC filters was used for fluorescence imaging and data collection to accurately measure ROS activity inside the cells. As a positive control, *tert*‐butyl hydroperoxide (TBHP) was used on the day of the experiment at a working concentration of 100 μM diluted in complete growth media, to induce ROS production. The TBHP solution was perfused into the tissue compartment containing TNBC cells and incubated for 1.5 h at 37°C. Subsequently, the cells were washed with HBSS, followed by the previously described H2DCFDA labeling method. Quantification was performed using ImageJ and cellSens to obtain RawIntDensity values, followed by statistical analysis.

### 2.10 Quantifying tumor intravasation

The intravasation assay was performed to evaluate TNBC transendothelial migration from the tissue compartment into the vascular channels, with or without PKCδ‐TAT treatment. The iMVoC were imaged daily, and endpoint snapshots were used to capture cell distribution across compartments. Quantification was performed using the *Count and Measure* feature of cellSens Dimension software, which enabled identification and enumeration of CM DiI‐labeled TNBC cells across three regions: the tissue compartment, the porous interface, and the vascular channels. The percentage of intravasated cells was calculated as the fraction of cells detected in the vascular compartment relative to the total. A detailed description and validation of this image analysis protocol have been reported previously^27^ and was applied here without modification.

### 2.11 Probing cytokine/chemokine production

Soluble factors secreted from the primary TME, particularly inflammatory cytokines, were analyzed to determine their role in TNBC metastasis. The microfluidic model was utilized for collecting soluble factors from each compartment following each treatment; media samples were collected from two vascular channels, centrifuged to get rid of any debris, and the samples were stored at −80°C until ready to use. These samples were analyzed using the Proteome Profiler Kits to assess a panel of pro‐inflammatory cytokines to determine the contribution of ECs in cytokine production. All reagents were brought to room temperature while samples were kept on ice. The nitrocellulose membrane pre‐spotted with capture antibodies for various cytokines, was blocked followed by incubation with prepared supernatant samples overnight at 4°C on a rocking platform. The next day, the membranes were carefully transferred to individual plastic containers containing 20 mL of 1× wash buffer, rinsed for 10 min, and the same process was repeated twice. Detection antibody was added, and membranes were incubated for 1 h on a rocking platform followed by washing. Then 1× streptavidin‐HRP was added to the membranes and incubated for 30 min at room temperature, followed by thorough washing. Chemiluminescent detection was performed by using prepared Chemi Reagent evenly for 1 min, removing the excess reagent and sealing with plastic wrap, ensuring no air bubbles, and placing in an autoradiography cassette for x‐ray film exposure (1–10 min, with multiple exposure times). Images were post‐processed and quantified using ImageJ imaging software.

### 2.12 Image quantification and statistical analysis

A range of software tools and statistical methods was utilized for image quantification, data analysis, and interpretation. The permeability and beads assays were analyzed using cellSens Dimensions and Microsoft Excel; the intravasation assay was quantified via cellSens Count and Measure alongside Excel. Oxygen levels were mapped using MATLAB, and the translocation of HIF‐1α and HIF‐2α proteins and ROS intensity was quantified using ImageJ software. The cytokine/chemokine data were acquired using a chemiluminescence reader, quantified and analyzed using ImageJ.

Unless otherwise stated in the figure legends, all experiments were performed with at least three independent biological replicates (*n* = 3). Data are presented as mean ± standard error of the mean (SEM), with individual data points shown in scatter plots. Statistical significance was determined using one‐way or two‐way analysis of variance (ANOVA) followed by Tukey's post hoc test for multiple comparisons (GraphPad Prism). A significance level of *α* = 0.05 was applied.

## 3 RESULTS

### 3.1 The hypoxic iMVoC model was successfully created using microfluidic device

**FIGURE 1:**
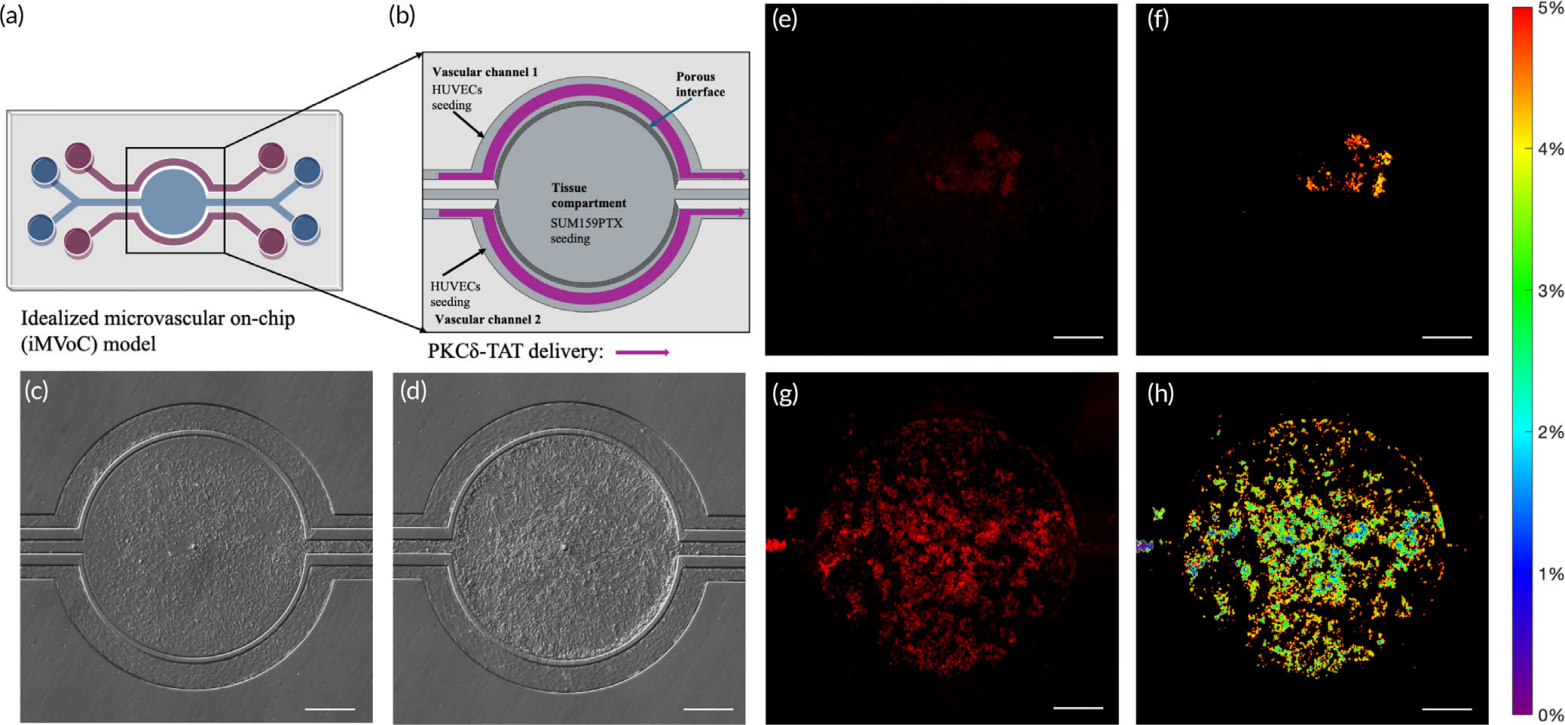
The hypoxic idealized microvascular on‐chip (iMVoC) model recapitulates tumor–endothelial cell (EC) interactions and hypoxic tumor microenvironment (TME). (a) Schematic of the hypoxic iMVoC with eight inlets/outlets and a central tissue compartment flanked by vascular channels. ECs were seeded in the vascular channels (purple, 200 μm wide, 100 μm height), while triple‐negative breast cancer (TNBC) cells were seeded in the tissue compartment (blue, 1.8 mm diameter, 100 μm height). (b) Close‐up view showing two semicircular vascular channels flanking the tissue compartment, separated by a porous microfabricated interface to support tumor‐EC co‐culture and mass exchange. The porous region consisted of a 50 μm‐wide gap spanned by an array of 10 μm‐diameter PDMS micropillars spaced 8 μm apart, providing structural support while permitting fluid exchange and cell migration between the compartments. (c, d) Bright‐field images of SUM159PTX‐HUVEC co‐culture at 0 h (c) and 48 h (d) post‐TNBC seeding. Vascular channels received continuous media perfusion, while the tissue compartment was bolus‐fed every 24 h. TNBC cells were embedded in 15% Matrigel to promote 3D spheroid formation. (e, f, g, h) Hypoxia mapping using Image‐iT Red (5 μM), a dye activated at <5% O_2_. Fluorescence at 24 h (e) and 96 h (g) was converted to oxygen concentration maps (f, h) using a calibration curve derived from sodium sulfite treated media and validated by DO meter. At 24 h, the tissue compartment remained largely normoxic, while significant hypoxia developed at 96 h due to increased tumor mass. The spatial oxygen gradient mimics the heterogeneous perfusion typical of solid tumors. Scale bars: 400 μm. HUVECs, human umbilical vein endothelial cells); PKCδ‐TAT, protein kinase C delta (PKCδ) inhibitor.

The hypoxic iMVoC model was successfully established using a microfluidic device designed with a porous interface to support TNBC–endothelial co‐culture (Figure 1a,b). TNBC cells embedded in 15% Matrigel were seeded into the tissue compartment, promoting 3D spheroid growth and restricted oxygen diffusion, while HUVECs were seeded into the vascular channels and maintained under continuous perfusion to form a functional endothelial barrier (Figure 1c,d). This configuration created a physiologically relevant hypoxic TME that enabled direct investigation of tumor–endothelial interactions.

As TNBC cells proliferated within the confined 3D tissue compartment, oxygen consumption outpaced diffusion from the adjacent vascular channels, resulting in the spontaneous formation of localized hypoxia (Figure 1e–h). Fluorescence hypoxia mapping revealed a spatial oxygen gradient, with stronger hypoxia signals (lower oxygen concentration) in the tumor core and weaker signals (higher oxygen concentration) at the periphery near the vascular interface (Figure 1h). Together, these results indicate that our model effectively replicates a hypoxic TME, wherein tumor cells experience oxygen deprivation similar to that found in aggressive solid tumors. The integration of fluorescence‐based hypoxia visualization and quantification provides a robust approach for analyzing oxygen dynamics in the hypoxic iMVoC model, allowing for further investigations into hypoxia‐driven tumor progression, endothelial activation, and therapeutic responses.

### 3.2 PKCδ inhibition suppresses HIF‐1α and HIF‐2α nuclear translocation in the hypoxic iMVoC model

**FIGURE 2:**
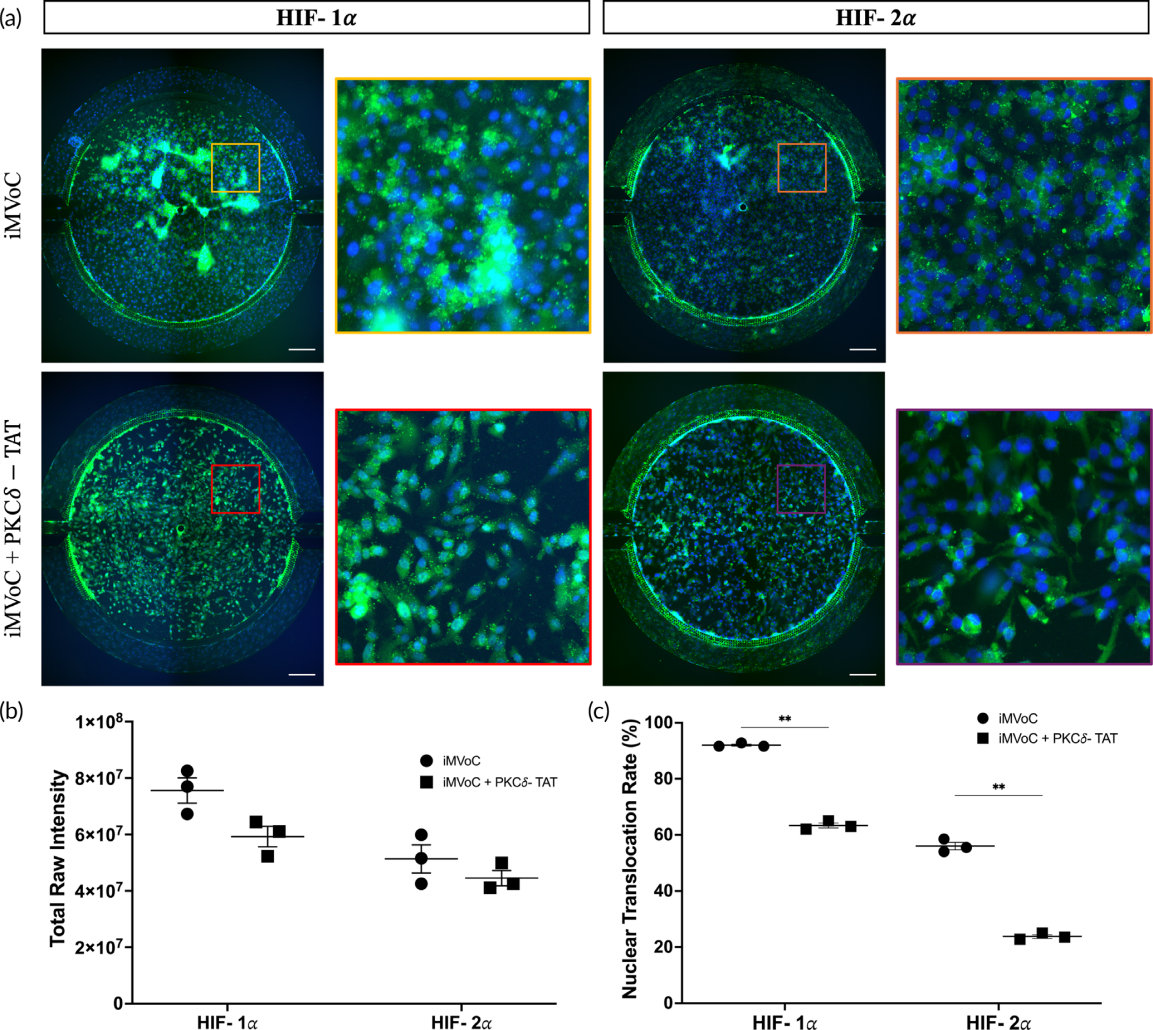
Protein kinase C delta (PKCδ) inhibition reduces hypoxia‐driven hypoxia inducible factors (HIF) activation and nuclear translocation in the hypoxic idealized microvascular on‐chip (iMVoC) model. (a) Representative immunofluorescence images of HIF‐1α and HIF‐2α expression in SUM159PTX 3D tumor structures within the tissue compartment. Green fluorescence indicates hypoxic regions with nuclear HIF accumulation. PKCδ inhibition by PKCδ‐TAT treatment reduces HIF disperses localization, suggesting restored oxygen homeostasis. Zoomed‐in inset panels highlight subcellular localization differences between untreated and PKCδ‐TAT treated conditions. (b) Quantification of total HIF fluorescence intensity shows no significant difference between groups. (c) Quantification of HIF subcellular localization shows significantly higher nuclear translocation for both HIF‐1α and HIF‐2α in untreated iMVoC, compared to those treated with PKCδ‐TAT (*p* < 0.01), indicating suppression of hypoxia‐induced signaling. **p < 0.01, by ANOVA. Scale bars: 200 μm.

We next assessed HIF‐1α and HIF‐2α localization in the hypoxic iMVoC model using fluorescence immunostaining and quantitative analysis (Figure 2). Under hypoxic conditions, both transcription factors exhibited strong nuclear accumulation, with ~90% of HIF‐1α and ~60% of HIF‐2α localized to the nucleus, accompanied by reduced cytoplasmic retention. Treatment with PKCδ‐TAT markedly altered this distribution, decreasing nuclear localization to ~55% for HIF‐1α and ~30% for HIF‐2α (*p* < 0.01). These findings indicate that PKCδ inhibition reduces hypoxia‐induced nuclear translocation of HIF‐1α/2α and promotes cytoplasmic retention, suggesting a role for PKCδ in facilitating HIF signaling under hypoxic conditions.

### 3.3 PKCδ inhibition reduces ROS generation in the hypoxic iMVoC model

**FIGURE 3:**
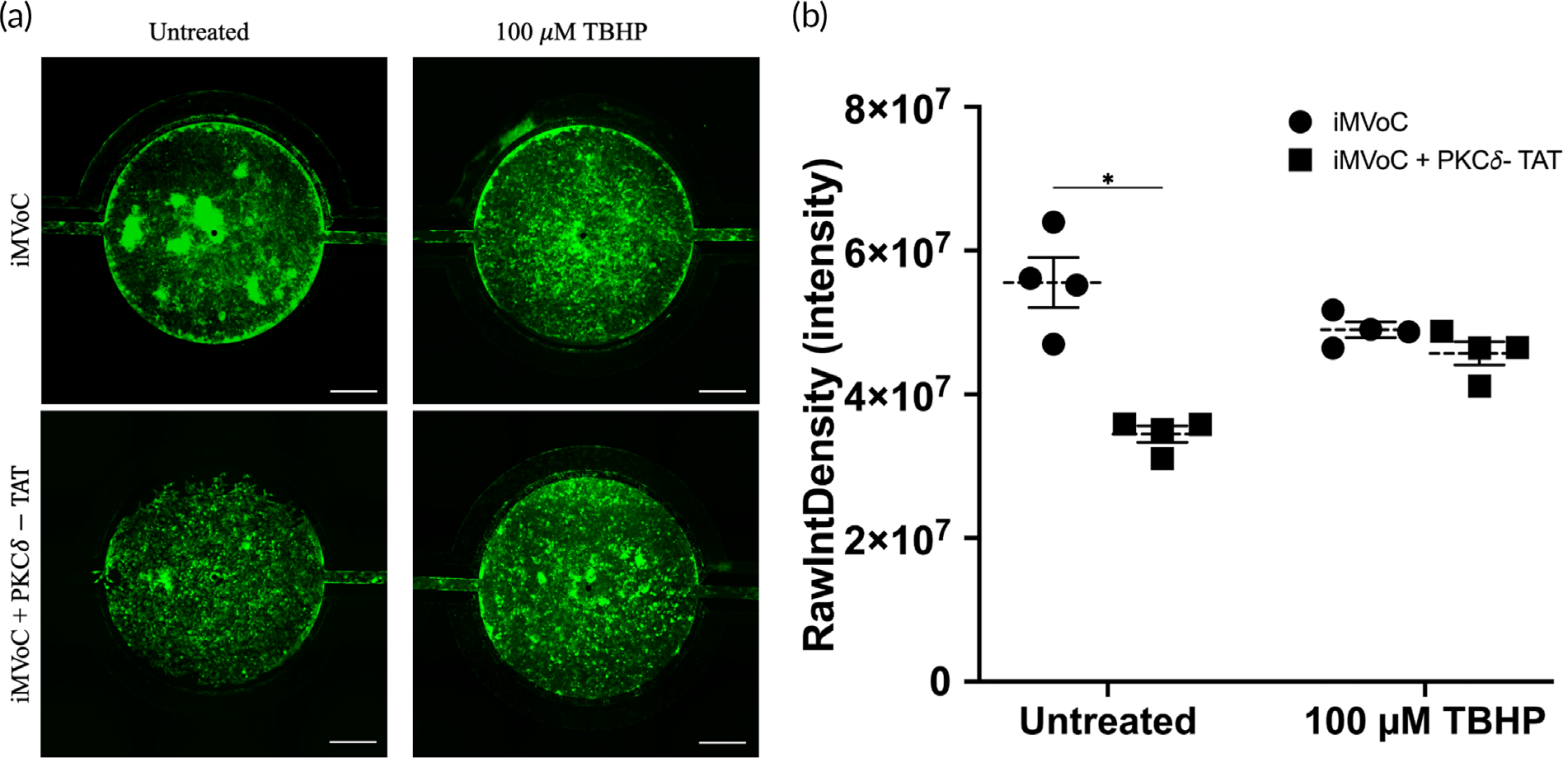
Protein kinase C delta (PKCδ) inhibition attenuates hypoxia‐associated reactive oxygen species (ROS) accumulation in the idealized microvascular on‐chip (iMVoC) model. (a) Representative fluorescence images showing ROS levels in triple‐negative breast cancer (TNBC) cells, stained with carboxy‐H_2_DCFDA, a ROS‐sensitive dye. *tert*‐Butyl hydroperoxide (TBHP) (100 μM), a pro‐oxidant that generates intracellular ROS via peroxyl and alkoxyl radical formation, was used as a positive control. Strong fluorescence in untreated iMVoC (top left), comparable to TBHP‐treated ones (top right), indicates elevated ROS under hypoxic conditions. PKCδ‐TAT treatment (bottom left) markedly reduces ROS signal intensity, suggesting attenuation of oxidative stress. In contrast, PKCδ‐TAT did not mitigate the ROS surge induced by TBHP (bottom right), consistent with the non‐specific, chemical nature of TBHP‐mediated oxidative stress. (b) Quantification of total fluorescence intensity (raw integrated density) confirms significantly lower ROS levels in PKCδ‐TAT‐treated iMVoC compared to untreated ones (*p* < 0.05). No significant difference was observed in TBHP‐treated iMVoC with or without PKCδ‐TAT, consistent with its non‐enzymatic ROS induction mechanism. *p < 0.05, by ANOVA. Scale bars: 400 μm.

To evaluate the effect of ROS production in the hypoxic iMVoC model, we measured intracellular ROS levels in TNBC cells using the fluorescent probe Carboxy‐DCFDA. Fluorescence imaging (Figure 3a) and quantitative analysis (Figure 3b) revealed increased basal ROS generation in untreated cultures, whereas PKCδ inhibition significantly reduced ROS levels (*p* < 0.05), indicating that PKCδ contributes to baseline ROS production under hypoxic conditions. To further assess the oxidative stress response, both groups were treated with the ROS inducer TBHP (100 μM). Following TBHP treatment, ROS levels were similarly elevated in both untreated and PKCδ‐TAT treated cultures, demonstrating that PKCδ inhibition does not alter exogenous ROS induction (Figure 3b). These results suggest that PKCδ specifically regulates basal, hypoxia‐driven ROS production but does not directly influence ROS generated by acute oxidative stressors. Overall, PKCδ inhibition may protect against chronic ROS accumulation in the TME, potentially mitigating oxidative stress‐induced endothelial dysfunction and tumor progression.

### 3.4 PKCδ inhibition preserves endothelial barrier integrity in the hypoxic iMVoC model

**FIGURE 4:**
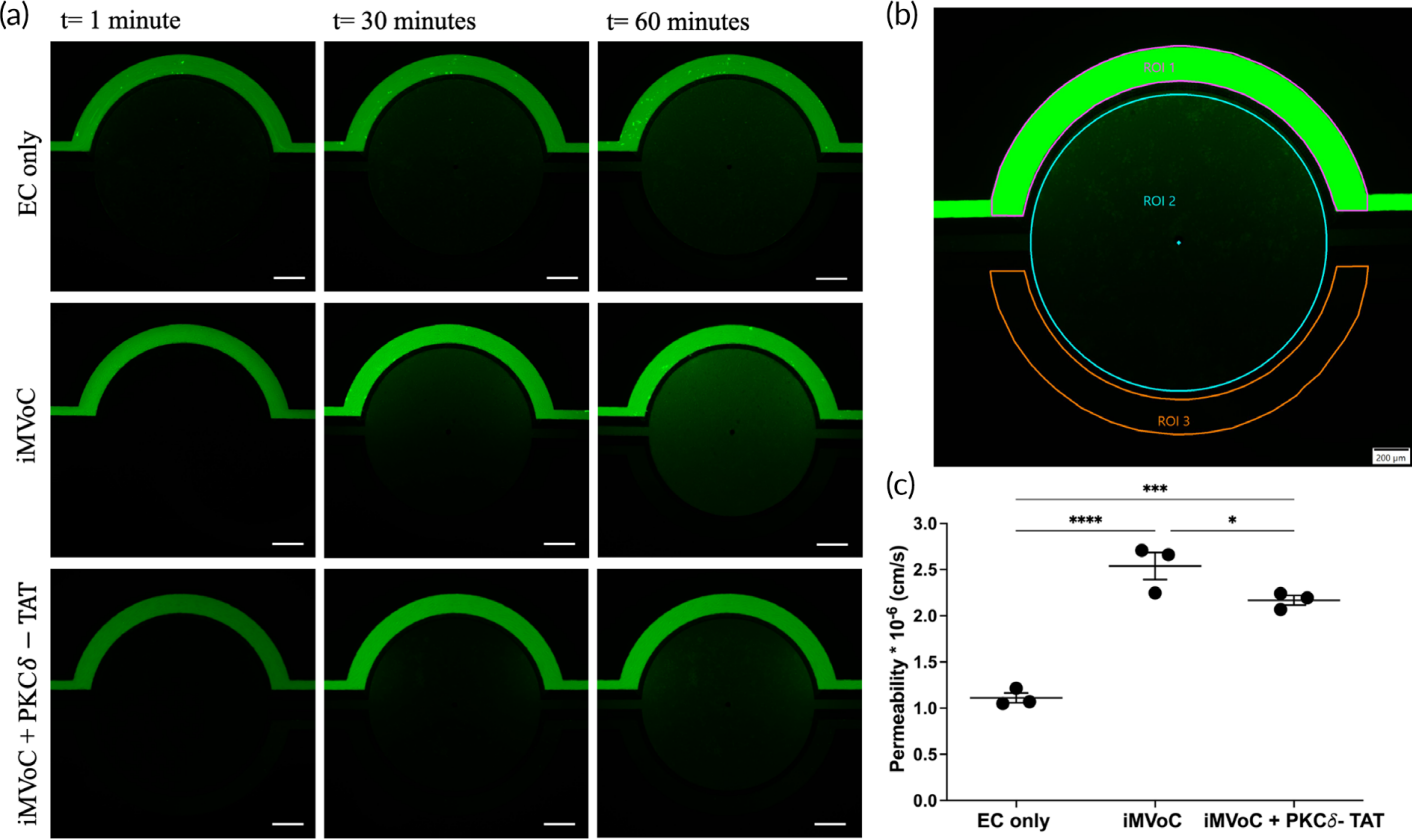
Protein kinase C delta (PKCδ) inhibition preserves endothelial barrier integrity in the hypoxic idealized microvascular on‐chip (iMVoC) model, as assessed by permeability assay. The permeation of 4 kDa FITC dextran dye from vascular channels to the tissue compartment was assessed by measuring fluorescent intensity of the dextran in the tissue compartment relative to the vascular channels over time. (a) Representative fluorescence images of the endothelial cells (EC)‐only, iMVoC, and iMVoC + PKCδ‐TAT groups at 1, 30, and 60 min. (b) Fluorescence intensity was measured in defined region of interest (ROIs): ROI 1 (vascular channel under flow), ROI 2 (tissue compartment), and ROI 3 (non‐flow vascular channel, background). (c) Quantification of permeability coefficients (cm/s × 10^−6^) across conditions showed significantly increased dextran diffusion in the untreated iMVoC compared with PKCδ‐TAT treated ones indicating compromised barrier function. *p < 0.05, ***p < 0.001 and ****p < 0.0001 by ANOVA. Scale bars: 200 μm.

Permeability assays (Figure 4) were performed using fluorescently labeled FITC dextran (4 kDa) perfused at 1 μL/min (1.75 dyne/cm^2^) for 1 h, with time‐lapse imaging collected at 1‐min intervals. Representative images of dextran diffusion from the vascular channels into the tissue compartment at 1, 30, and 60 min are shown in Figure 4a for EC‐only (no tumor), untreated iMVoC, and PKCδ‐TAT treated iMVoC groups. ROIs used for quantification are illustrated in Figure 4b. Quantitative analysis (Figure 4c) demonstrated that the EC‐only control maintained a stable endothelial barrier with a permeability value of (1.10 ± 0.0421) × 10^−6^ cm/s. In contrast, the iMVoC group exhibited a significant increase to (2.53 ± 0.147) × 10^−6^ cm/s compared with EC‐only controls (*p* < 0.0001), confirming that tumor‐EC interactions compromise vascular integrity. PKCδ‐TAT treatment partially restored barrier function, reducing permeability to (2.1 ± 0.078) × 10^−6^ cm/s compared with untreated iMVoC (*p* < 0.05). Although barrier integrity improved, the intervention did not fully reverse tumor‐induced endothelial dysfunction. These findings are consistent with earlier results, where PKCδ inhibition attenuated hypoxia‐driven HIF nuclear translocation (Figure 2) and reduced ROS production in TNBC cells (Figure 3), both of which contribute to endothelial barrier disruption.

### 3.5 PKCδ inhibition attenuated hypoxia‐induced endothelial adhesion molecule expression in the hypoxic iMVoC model

**FIGURE 5:**
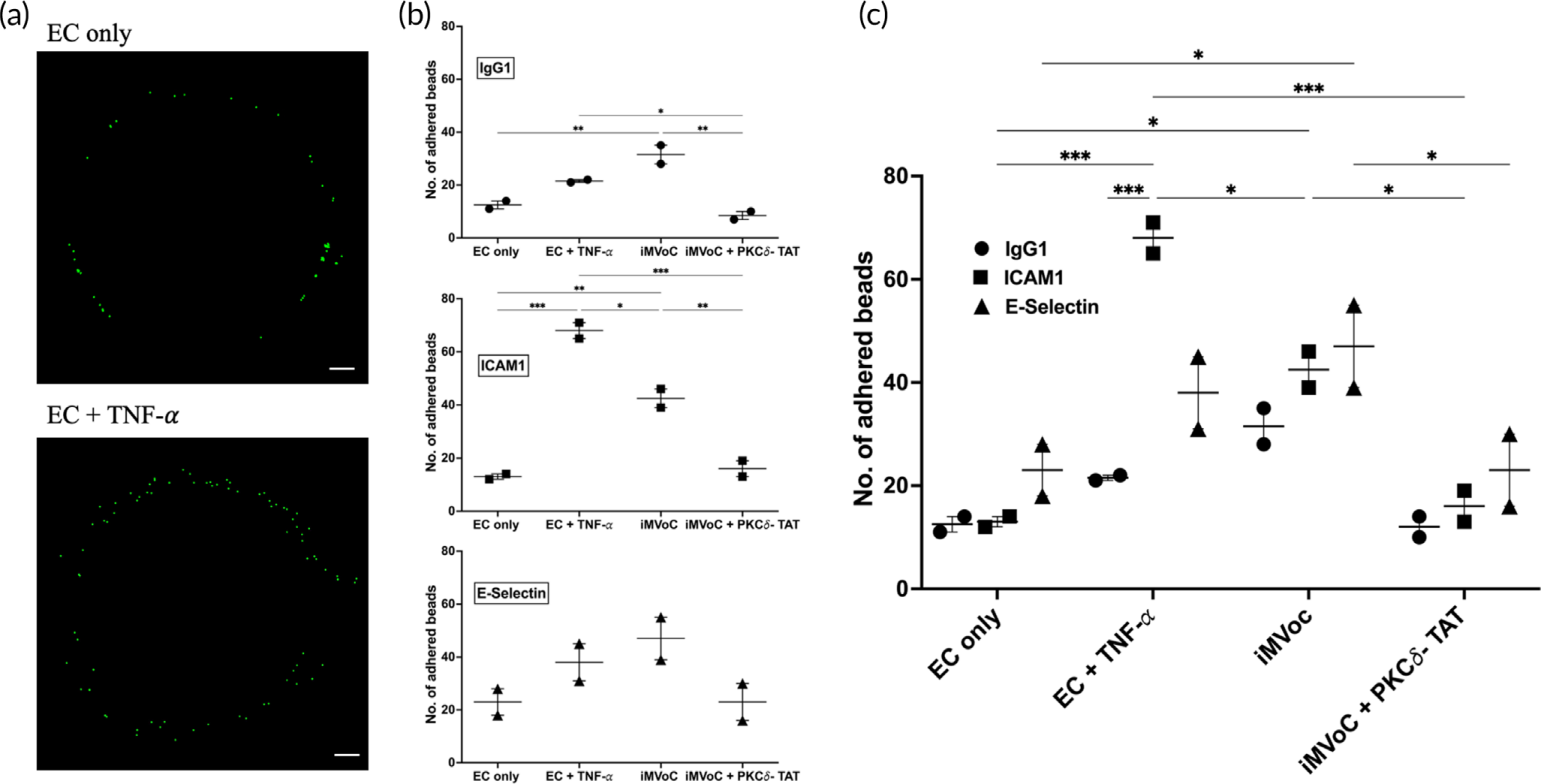
Protein kinase C delta (PKCδ) inhibition attenuates adhesion molecule upregulation in the hypoxic idealized microvascular on‐chip (iMVoC) model. (a) Representative fluorescence images from microbead adhesion assays using polystyrene beads coated with antibodies against IgG1 (isotype control), ICAM‐1, or E‐selectin. Beads were introduced under continuous flow (1.75 dyne/cm^2^) to assess endothelial activation and adhesion molecule expression. (b, c) Quantification showed marked upregulation of E‐selectin and ICAM‐1 in untreated iMVoC cultures. PKCδ‐TAT treatment suppressed this response, reducing adhesion molecule levels toward baseline. *p < 0.05, **p < 0.01, ***p < 0.001, by ANOVA. Scale bars: 200 μm. EC, endothelial cells.

Activated endothelium upregulates adhesion molecules such as ICAM‐1 and E‐selectin, which facilitate tumor cell adhesion and intravasation.^36^, ^37^ To assess endothelial activation, we performed a microbead adhesion assay with IgG1‐, ICAM‐1‐, and E‐selectin‐coated microbeads under flow (1.75 dyne/cm^2^) (Figure 5). Tumor necrosis factor‐alpha (TNF‐α), a key inflammatory cytokine that enhances endothelial activation, vascular permeability, and tumor–endothelial interactions,^38^, ^39^, ^40^ was included as a positive control. The hypoxic iMVoC model showed robust EC activation with increased bead adhesion, particularly via ICAM‐1, though less pronounced than TNF‐α stimulation. As expected, TNF‐α significantly upregulated adhesion across all bead types compared to EC‐only controls (*p* < 0.001). PKCδ‐TAT treatment reduced adhesion by ~30% to 40% relative to untreated iMVoC, with the strongest effect on ICAM‐1‐mediated binding. Statistical analysis confirmed significant differences across all groups (*p* < 0.05–0.001). These findings demonstrate that tumor–endothelial interactions in the hypoxic iMVoC model promote endothelial activation, while PKCδ inhibition attenuates ICAM‐1 and E‐selectin expression, thereby reducing adhesion. In line with prior results showing that PKCδ inhibition disrupts HIF signaling (Figure 2), decreases ROS accumulation (Figure 3), and stabilizes barrier function (Figure 4), these data further establish its anti‐inflammatory role in the hypoxic TME.

### 3.6 PKCδ inhibition reduced TNBC intravasation from the tissue compartment into the vascular channels in the hypoxic iMVoC model

**FIGURE 6:**
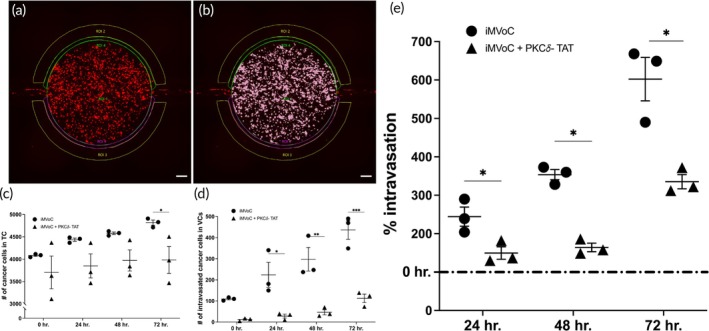
Protein kinase C delta (PKCδ) inhibition reduces triple‐negative breast cancer (TNBC) intravasation across the endothelial barrier in the hypoxic idealized microvascular on‐chip (iMVoC) model. (a, b) Representative endpoint images of the iMVoC before (a) and after (b) analysis using the “Count and Measure” function to track cancer cell distribution across compartments. (c) Total TNBC cell count in the tissue compartment (TC) remains stable over time, indicating no treatment‐induced cytotoxicity. (d, e) Quantification of intravasated SUM159PTX cells in the vascular channels (VCs), shown as absolute counts (d) and percent increase over baseline (e). Intravasation significantly increases over time in untreated iMVoC cultures, while PKCδ‐TAT treatment markedly suppresses transendothelial migration at all time points. *p < 0.05, **p < 0.01, ***p < 0.001, by ANOVA. Scale bars: 200 μm.

To assess tumor cell intravasation, CM‐DiI‐labeled TNBC cells were tracked in the tumor compartment and vascular channels using fluorescence imaging and time‐lapse microscopy (Figure 6a,b). Intravasated cells were first detected at 24 h post‐seeding and increased significantly at 48 and 72 h (Figure 6d). In untreated iMVoC, hypoxia and inflammation promoted tumor–endothelial interactions and transendothelial migration. PKCδ inhibition markedly reduced the number of intravasated TNBC cells at all time points (*p* < 0.05), indicating that PKCδ contributes to endothelial remodeling and tumor cell migration into the vasculature. Importantly, the total number of TNBC cells in the tumor compartment remained comparable between groups (Figure 6c), showing that PKCδ inhibition did not impair tumor cell proliferation or viability. To normalize for cell population, the percentage of intravasated cells was calculated relative to the total TNBC population in the tissue compartment (Figure 6e). This percentage was consistently lower in the PKCδ‐TAT treated group compared to untreated iMVoC at 24, 48, and 72 h (*p* < 0.05), confirming that PKCδ inhibition specifically limits TNBC intravasation in the hypoxic iMVoC model.

### 3.7 PKCδ inhibition reduces pro‐inflammatory cytokine and chemokine expression

**FIGURE 7:**
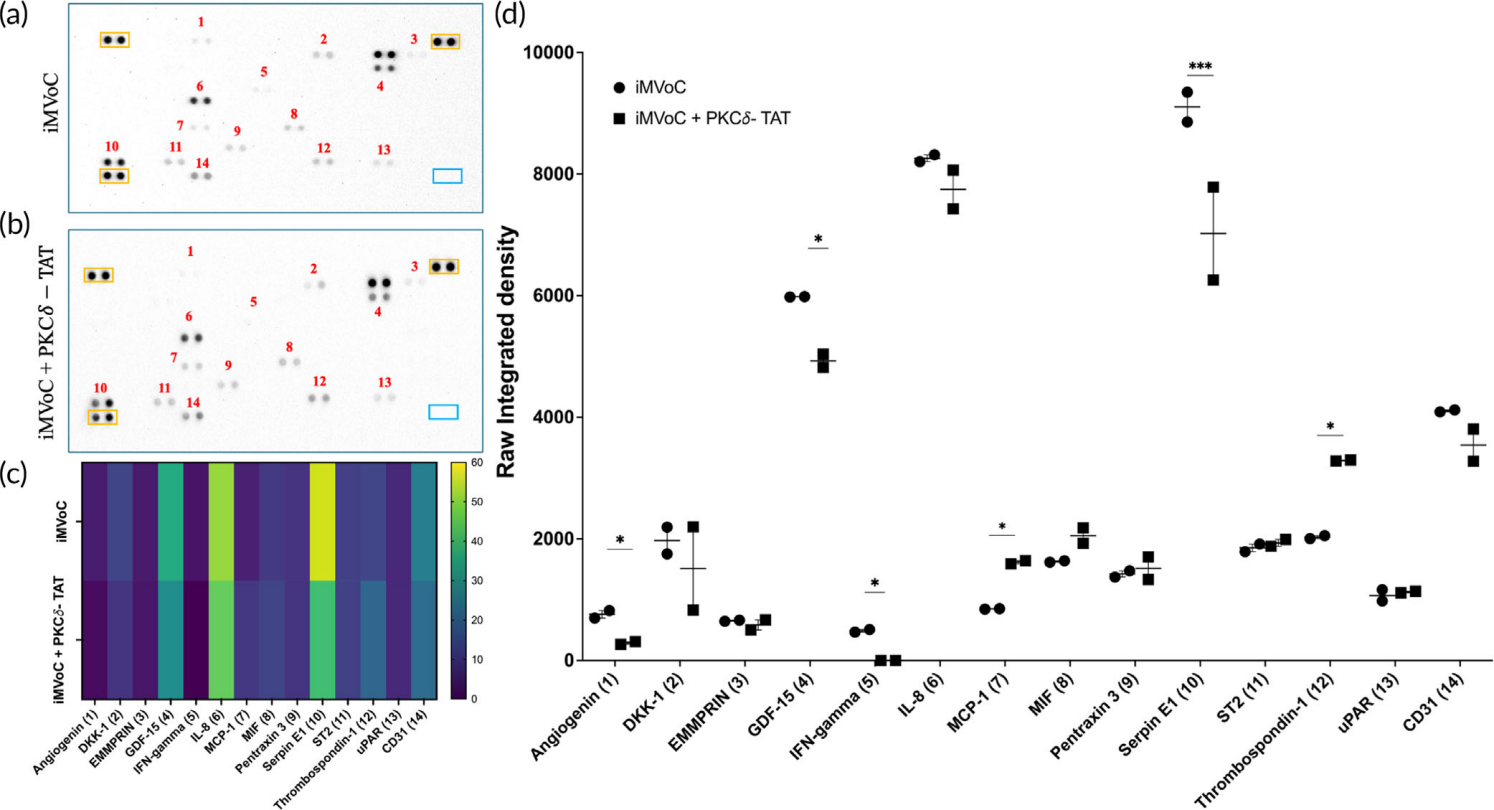
Protein kinase C delta (PKCδ) inhibition modulates cytokine secretion in the hypoxic idealized microvascular on‐chip (iMVoC) model. (a, b) Representative cytokine array images of conditioned media collected from the vascular channel of (a) untreated and (b) PKCδ‐TAT treated iMVoC. Each cytokine was detected in duplicate and numerically labeled for reference. (c) Heatmap showing fold changes in cytokine expression between treatment groups. (d) Quantified signal intensities (raw integrated density) demonstrate that PKCδ‐TAT treatment significantly reduced the secretion of several pro‐inflammatory and metastasis‐related cytokines, including Angiogenin, GDF‐15, IFN‐γ, and serpin E1 (*p < 0.05, ***p < 0.001 by ANOVA). In contrast, MCP‐1 and Thrombospondin‐1 levels were increased, indicating selective modulation of signaling within the hypoxic iMVoC model.

To evaluate how hypoxic TNBC–endothelial interactions shape the secretome, conditioned media from the vascular channels of iMVoC cultures were analyzed using a cytokine array. Fourteen cytokines were consistently detectable across treatment groups (Figure 7a,b). These factors could be broadly classified into three functional categories: (i) pro‐inflammatory and chemotactic mediators (interleukin‐8 [IL‐8], interferon‐gamma [IFN‐γ], monocyte chemoattractant protein‐1 [MCP‐1], macrophage migration inhibitory factor [MIF], and interleukin 1 receptor‐like 1 [ST2]), which promote leukocyte recruitment and endothelial activation; (ii) angiogenic and vascular remodeling factors (angiogenin, growth differentiation factor 15 [GDF‐15], extracellular matrix metalloproteinase inducer [EMMPRIN], urokinase plasminogen activator surface receptor [uPAR], and CD31), which stimulate neovascularization and matrix degradation; and (iii) extracellular matrix (ECM) modulators and regulatory proteins (serpin E1, pentraxin‐3, dickkopf‐1 [DKK‐1], and thrombospondin‐1), which influence ECM remodeling, Wnt signaling, and anti‐angiogenic responses. In the hypoxic iMVoC model, multiple pro‐inflammatory and angiogenic mediators including angiogenin, GDF‐15, IFN‐γ, and serpin E1 were elevated (Figure 7c,d), consistent with a metastasis‐permissive phenotype. PKCδ‐TAT treatment significantly attenuated this profile, suppressing secretion of angiogenin, GDF‐15, IFN‐γ, and serpin E1. In contrast, MCP‐1 and thrombospondin‐1 were upregulated following PKCδ inhibition. Although both are often associated with inflammation, they have also been implicated in vascular maturation, tissue repair, and anti‐angiogenic signaling, suggesting a context‐dependent, compensatory reprogramming of the secretome under PKCδ blockade (see Section 4). Collectively, these results indicate that PKCδ inhibition shifts the hypoxic secretory profile away from pro‐inflammatory and pro‐angiogenic factors toward a more regulated state, thereby linking hypoxia‐driven cytokine signaling, endothelial dysfunction, and TNBC intravasation.

## 4 DISCUSSION

This study builds upon our previously published microfluidic tumor model^27^ but introduces key innovations to investigate hypoxia‐driven endothelial dysfunction and TNBC intravasation. Compared to global hypoxia models that expose all compartments to uniform oxygen deprivation (e.g., hypoxia chambers), our approach enables spontaneous hypoxia formation in the tumor compartment of iMVoC while maintaining ECs under normoxia via media perfusion. This configuration more closely mimics the in vivo situation, in which ECs respond to tumor‐secreted factors and hypoxia‐driven cross‐compartmental signaling rather than direct, sustained hypoxia. The geometry of the iMVoC device was carefully selected to balance physiological relevance with experimental feasibility: the tissue compartment diameter of 1.8 mm supports the formation of 3D tumor spheroids exceeding 500 μm, a threshold at which proliferative, quiescent, and necrotic zones emerge due to diffusion limitations, thereby reproducing the heterogeneity and hypoxia of solid tumors in vivo.^41^ The vascular channels, measuring 200 μm wide and 100 μm high, provide appropriate dimensions for endothelial monolayer formation, flow‐mediated alignment, and barrier integrity, as demonstrated by us and others.^27^, ^42^, ^43^, ^44^, ^45^ These design choices ensure that the model not only permits spontaneous hypoxia formation in the tumor compartment but also maintains reproducibility and clear visualization of endothelial responses. By enabling compartment‐specific hypoxia alongside quantitative analysis of HIF translocation, cytokine signaling, and ROS production, the model enhances physiological relevance and provides new mechanistic insight into how hypoxia destabilizes the vasculature. These advances underscore the innovation and significance of the iMVoC platform as a tool to dissect tumor–endothelial interactions and evaluate strategies such as PKCδ inhibition to suppress TNBC metastasis.

Previous in vitro studies have extensively demonstrated the role of hypoxia in altering EC phenotype. For example, chronic hypoxia decreases CD31 expression, promoting vascular permeability and inflammation.^46^, ^47^ Prolonged hypoxia (>24 h) induces EC senescence, characterized by flattened morphology and increased membrane stiffness, which impairs structural integrity.^46^, ^48^ Other studies reported enhanced ICAM‐1 expression via HIF‐1α‐arginase (Arg‐II)‐mitochondrial ROS signaling, leading to increased monocyte adhesion, along with upregulation of E‐selectin that facilitates neutrophil recruitment.^49^ Hypoxia has also been shown to shift ECs toward an inflammatory phenotype through HIF‐dependent pathways, elevating IL‐8 and disrupting vascular networks.^50^ In addition, hypoxia promotes a metabolic switch in ECs that enhances angiogenesis through lactate production, HIF stabilization, and endothelial sprouting.^51^ Collectively, these studies highlight hypoxia‐induced EC dysfunction; however, the direct mechanistic link to tumor metastasis remains unclear. Specifically, how hypoxia‐driven EC activation supports tumor cell intravasation and metastatic progression within the TME has yet to be fully elucidated.

This study builds on previous findings that maintaining endothelial integrity reduces TNBC intravasation^27^ by examining the upstream factors contributing to endothelial dysfunction, particularly the influence of hypoxia on the tumor–ECs interface through a modified microfluidic co‐culture model. In this study, we sought to systematically investigate the interplay between hypoxia, endothelial activation, and TNBC intravasation using a hypoxic iMVoC microfluidic model that simulates tumor–EC interactions. This platform integrates vascular perfusion and allows for real‐time analysis of TNBC–ECs interactions. The hypoxic iMVoC model developed in this study enabled the simultaneous co‐culture of ECs in vascular channels under physiological shear flow (0–1.75 dyne/cm^2^), whereas TNBC cells were seeded in the tissue compartment (Figure 1c,d), which facilitates the formation of hypoxia. The creation of hypoxia in TNBC cells in the tissue compartment was confirmed using an oxygen‐sensitive fluorescent dye and its mapping as shown in Figure 1e–h. That enabled an investigation into the effects of the hypoxic TME in ECs and its effects on TNBC intravasation.

Endothelial barrier integrity is essential for maintaining vascular homeostasis and regulating the exchange of fluids, solutes, and cells between the bloodstream and surrounding tissues.^52^, ^53^ A compromised barrier is frequently observed in pathological contexts such as inflammation, tumor progression, and metastasis, where increased permeability facilitates tumor cell extravasation and immune infiltration.^54^ Within the TME, hypoxia and oxidative stress are major drivers of endothelial dysfunction, leading to disruption of vascular integrity and amplification of permeability.^55^ In our hypoxic iMVoC model, endothelial permeability was significantly elevated (Figure 4) and accompanied by increased ROS production (Figure 3), implicating oxidative stress as a central mediator of barrier disruption. Functionally, these changes translated into markedly higher rates of TNBC intravasation across endothelial barriers compared to PKCδ‐TAT‐treated iMVoC (Figure 6). Mechanistic analyses revealed that hypoxia‐induced pronounced nuclear localization of HIF‐1α and HIF‐2α in TNBC cells, reflecting activation of transcriptional programs that exacerbate endothelial dysfunction (Figure 2). Concurrently, endothelial activation was evidenced by strong upregulation of adhesion molecules, including ICAM‐1 and E‐selectin (Figure 5). Notably, ICAM‐1 expression equaled or exceeded that induced by TNF‐α, a potent cytokine stimulus, suggesting a substantial inflammatory burden in the iMVoC. Collectively, these findings define a hypoxia‐ROS‐HIF axis that destabilizes endothelial junctions, elevates permeability, and drives TNBC transendothelial migration.

PKCδ emerged as a pivotal regulator of this process. PKCδ is a central regulator of inflammation‐driven endothelial activation.^28^, ^29^, ^30^, ^31^, ^33^, ^34^, ^35^ Its activity is tightly controlled by site‐specific tyrosine phosphorylation, which dictates activation, localization, and substrate specificity.^56^, ^57^, ^58^ PKCδ‐TAT, a peptide derived from the V1 domain (residues 8–17: SFNSYELGSL), disrupts PKCδ docking interactions to block translocation and substrate binding, exerting anti‐inflammatory effects without general immunosuppression.^28^, ^59^, ^60^ Our prior in vitro and in vivo studies demonstrated that PKCδ‐TAT stabilizes endothelial junctions, reduces neutrophil extravasation, and downregulates adhesion molecules.^29^, ^30^, ^33^, ^34^, ^35^ Extending these findings to the hypoxic iMVoC model, PKCδ‐TAT treatment (5 μM, 72 h) consistently suppressed nuclear accumulation of HIF‐1α and HIF‐2α (Figure 2), reduced ROS generation (Figure 3), downregulated ICAM‐1 and E‐selectin (Figure 5), stabilized barrier function (Figure 4), and decreased TNBC intravasation (Figure 6). Importantly, PKCδ‐TAT did not rescue ROS induced by TBHP, which generates exogenous oxidative stress independent of PKCδ signaling, but it effectively reduced basal hypoxia‐driven ROS, linking PKCδ to HIF stabilization and endothelial dysfunction. Mechanistically, PKCδ modulates HIF‐1α stability by influencing its interaction with prolyl hydroxylases, thereby enhancing HIF‐1α accumulation and nuclear translocation.^61^ Once stabilized, nuclear HIF‐1α initiates transcription of pro‐inflammatory cytokines and chemokines, activating NF‐κB signaling.^62^, ^63^, ^64^ This cascade, amplified by ROS,^62^, ^63^ drives endothelial activation, vascular permeability, and tumor cell transendothelial migration. Taken together, our results identify PKCδ as a central molecular node that integrates hypoxic signaling, ROS generation, and inflammatory responses, and highlight the therapeutic potential of PKCδ inhibition to preserve vascular integrity and limit TNBC metastasis.

Our proteomic analysis (Figure 7) further supported our findings, highlighting elevated levels of pro‐inflammatory cytokines and chemokines in the untreated iMVoC model. Under hypoxic conditions, pro‐angiogenic factors such as angiogenin and GDF‐15 were upregulated, promoting neovascularization and vascular remodeling.^64^, ^65^ Inflammatory mediators including IL‐8, MCP‐1, IFN‐γ, MIF, and ST2 enhanced EC activation and adhesion molecule expressions, thereby exacerbating the inflammatory TME.^66^, ^67^, ^68^, ^69^ ECM‐modulating factors such as uPAR, EMMPRIN, Serpin E1, and thrombospondin‐1 further contributed to matrix remodeling and cancer cell invasion.^70^, ^71^, ^72^, ^73^ Additionally, DKK‐1 plays a regulatory role in Wnt signaling, influencing EC integrity,^74^ and CD31, a junctional adhesion molecule and marker of angiogenic ECs, was also detected, consistent with endothelial remodeling and angiogenesis under hypoxic conditions.^75^ Collectively, these changes reflect an angiogenic, pro‐inflammatory, and ECM‐remodeling phenotype consistent with endothelial dysfunction and a permissive environment for TNBC intravasation.

PKCδ‐TAT treatment significantly altered this cytokine profile. Six factors (angiogenin, IFN‐γ, MCP‐1, GDF‐15, serpin E1, and thrombospondin‐1) showed significant differences between untreated and PKCδ‐TAT groups. Most pro‐angiogenic and inflammatory signals (angiogenin, GDF‐15, IFN‐γ, and serpin E1) were reduced, consistent with dampened angiogenic and inflammatory signaling and reduced endothelial activation. Interestingly, MCP‐1 and thrombospondin‐1 were upregulated after PKCδ inhibition. MCP‐1, although classically pro‐inflammatory, has also been implicated in vascular normalization and tissue repair^76^ and in promoting EC maturation and vascular integrity under low‐inflammation conditions.^77^ Thrombospondin‐1, traditionally recognized as an anti‐angiogenic factor, also participates in context‐dependent tissue remodeling and endothelial regulation,^73^ and its anti‐angiogenic functions may suppress tumor progression.^78^, ^79^ Together, their elevation may represent a compensatory or regulatory response rather than a strictly pro‐metastatic signal. Overall, PKCδ inhibition reduced cytokine secretion, improved endothelial barrier function, and decreased tumor intravasation, establishing a mechanistic link between hypoxia‐driven endothelial activation, matrix remodeling, and TNBC intravasation.

**FIGURE 8:**
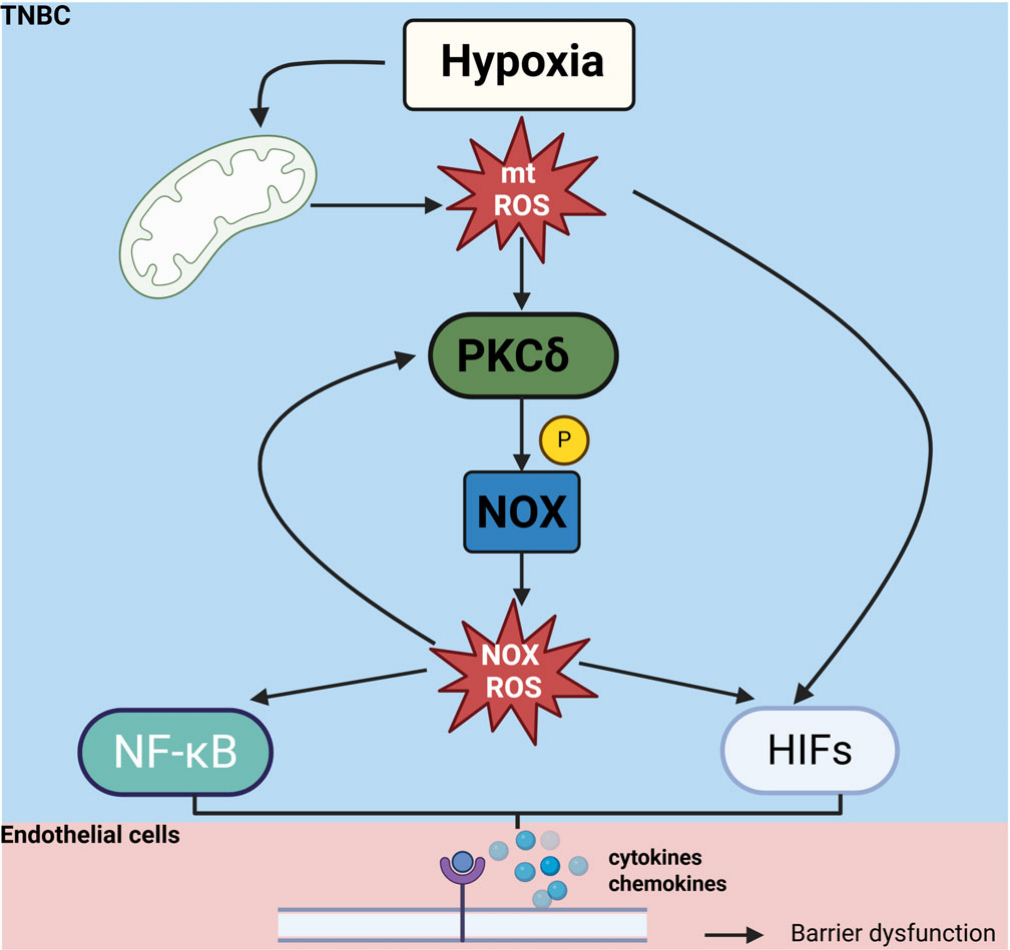
Mechanistic model of hypoxia‐induced protein kinase C delta (PKCδ) activation and endothelial dysfunction. Under hypoxic conditions, mitochondrial reactive oxygen species (mtROS) generated in triple‐negative breast cancer (TNBC) cells stabilize hypoxia inducible factors (HIF)‐1α/2α and activate PKCδ through redox‐sensitive phosphorylation. Activated PKCδ amplifies oxidative stress by stimulating NADPH oxidase (NOX), leading to sustained ROS accumulation. The resulting oxidative and inflammatory signaling activates NF‐κB, promoting cytokine and chemokine release that induce endothelial activation, adhesion molecule upregulation, and vascular barrier disruption. PKCδ‐TAT inhibits PKCδ activation, dampening the ROS‐HIF‐NF‐κB feed‐forward loop and preserving endothelial integrity. Created in BioRender.

To synthesize our findings, we propose a mechanistic framework illustrated in Figure 8. In the hypoxic iMVoC model, stabilization of HIF‐1α/2α in tumor cells is accompanied by increased ROS production and release of soluble mediators that diffuse into the vascular compartment. These signals act as extracellular oxidative and inflammatory cues that activate PKCδ in ECs, which in turn stimulates NF‐κB signaling, leading to upregulation of adhesion molecules, cytokine secretion, and barrier disruption that facilitate TNBC intravasation. PKCδ inhibition interrupts this cascade at multiple levels. In tumor cells, it suppresses ROS generation and reduces HIF‐1α/2α nuclear translocation. In ECs, it prevents PKCδ‐dependent NF‐κB activation, limits adhesion molecule expression, and preserves barrier integrity. This dual effect positions PKCδ as a nodal integrator linking hypoxia, oxidative stress, and inflammatory signaling between tumor and endothelial compartments, highlighting its therapeutic potential to restrain metastatic progression.

## 5 FUTURE DIRECTIONS

This study intentionally focused on tumor–endothelial interactions in order to isolate the direct impact of hypoxia and PKCδ signaling on endothelial dysfunction and intravasation. While this reductionist approach highlights a critical mechanism, the iMVoC model does not currently include stromal or immune components that also shape the TME. Incorporating additional cell types, such as fibroblasts and immune populations, into future iterations of the platform will help capture the full complexity of tumor‐vascular interactions. Moreover, complementary genetic approaches (e.g., PKCδ overexpression or knockdown) and in vivo validation will be important to confirm the causality of PKCδ signaling in endothelial activation and metastatic progression. Future studies will also include direct assessment of PKCδ phosphorylation under hypoxic conditions to confirm the mechanism of inhibition. In addition, dissecting the PKCδ‐HIF‐ROS‐cytokine axis in more detail will be important for clarifying how hypoxia‐driven endothelial dysfunction promotes TNBC intravasation and for identifying points of therapeutic intervention. These next steps will extend the mechanistic insights presented here and strengthen the translational relevance of PKCδ‐targeted strategies.

## CONCLUSION

In summary, our findings demonstrate that hypoxia‐induced endothelial activation, mediated in part by PKCδ signaling, contributes to enhanced TNBC intravasation. PKCδ inhibition preserved endothelial barrier integrity, reduced inflammatory signaling, and suppressed metastatic dissemination in our model, underscoring the mechanistic link between hypoxia, PKCδ activity, and endothelial dysfunction. Together, these results highlight the active role of the endothelium in TNBC metastasis and establish the hypoxic iMVoC platform as a valuable tool for dissecting tumor–vascular crosstalk.

## AUTHOR CONTRIBUTION

Indira Sigdel, Awurama Ofori‐Kwafo, and Earshed Al Mamun conducted the experiments. Indira Sigdel wrote the original draft of the manuscript. Yuan Tang and Amit K. Tiwari were responsible for conceptualization, data analysis, funding acquisition, provision of resources, and manuscript editing. Yuan Tang designed and supervised the entire study, organized experiments, and contributed to manuscript writing. All authors contributed to the article, reviewed the manuscript, and approved the submitted version.

## CONFLICT OF INTEREST STATEMENT

There is no conflict of interest to disclose.

## Data Availability

The data that support the findings of this study are available from the corresponding author upon reasonable request.
